# The Effect of Alcohol on the Fœtus through the Blood of the Mother

**Published:** 1883-10

**Authors:** 


### The Effect of Alcohol on the Fœtus, Through the Blood of the Mother.

Dr. W. H. McDaniel writes on this subject, as follows, to the *Maryland Den. Jour.*, May 19, 1883:

"My experience as a medical practitioner for the past twelve years has taught me that it requires a very small dose of alcohol to produce fatal results to infants or young children, and the fact that more infants are not still-born from maternal intoxication is really owing to the fact that such conditions of the mother, during parturition, are fortunately very rare. That cases of this nature, however, do happen, I have not the least doubt, as I have had at least eight in my own practice, which could not be accounted for in any other way. For instance, three years ago I was called about 6 o'clock one morning to a woman who had been put to bed the previous evening in a beastly state of intoxication. I found her still drunk, and unable to articulate so as to be easily understood when she attempted to speak. In the bed beside her lay a large, healthy-looking male child with the umbilical cord and placenta attached. There was nothing to indicate that the child died of anything else than alcoholic poisoning contracted by absorption from the mother's blood. The mother herself had no recollection afterwards how or when her offspring came into the world. The husband, who is a sober, industrious man, had slept in the same bed with his wife, but had no knowledge of the birth till just before he summoned me. In the case of prostitutes, we often find that the decomposed bodies of infants will come away from them at any time from the fourth to the ninth month, and still no syphilitic history nor attempts at abortion in the ordinary ways, can be found to explain the cause. A succession of alcoholic debauches might, I think, be very likely to cause the death of the fœtus in utero, just as surely as it is liable to be in the cases of those whose constitutions are calculated to resist the effects of so powerful a poison, with plenty of pure air and physical maturity to back them up. If the fœtus during its intra-uterine life is in any degree liable to suffer from alcoholic intoxication on the part of the mother, then how much more pernicious must the effects of the maternal intoxication be on the fœtus after birth, when it is altogether dependent on its own organs for existence? Dr. Carpenter speaks very plainly on this point, and leaves no room for argument to the contrary."

